# Virtual Reality Implementation in Mental Health Care Is a Marathon, Not a Sprint: Qualitative Longitudinal Study of a Virtual Reality Training Program

**DOI:** 10.2196/83453

**Published:** 2026-03-10

**Authors:** Marileen MTE Kouijzer, Laura AM Koenis, David Huizinga, Saskia M Kelders, Yvonne HA Bouman, Hanneke Kip

**Affiliations:** 1 Department of Technology, Human and Institutional Behaviour, University of Twente, Drienerlolaan 5, Enschede, 7522 NB, The Netherlands, 31 534899111; 2 Department of Research, Transfore, Deventer, The Netherlands; 3 Dimence Mental Health Care Centre, Deventer, The Netherlands; 4 Opentia Research Unit, North-West University, Vanderbijlpark, South Africa

**Keywords:** virtual reality, VR, implementation, training, mental health care, Theoretical Domains Framework, TDF, capability, opportunity, motivation – behavior model, COM-B, implementation strategy

## Abstract

**Background:**

Despite the potential of virtual reality (VR) for treatment and assessment in mental health care, its practical implementation remains limited. Much implementation research explores barriers and facilitators; fewer studies actually evaluate targeted implementation strategies and track how their effects evolve over time in mental health care practice.

**Objective:**

This study aims to examine how a structured VR training program functioned as an implementation strategy in routine mental health care and to identify how therapists’ adoption trajectories and implementation needs shifted across stages of the process.

**Methods:**

Eleven therapists from a Dutch mental health care organization completed a 6-session VR training. Semistructured interviews were conducted at 3 time points: pretraining, immediately posttraining, and 3 months posttraining. Data were deductively analyzed using theoretical thematic analysis based on the capability, opportunity, motivation – behavior model and the Theoretical Domains Framework to map stage-specific changes in implementation needs relating to VR use.

**Results:**

The training improved therapists’ perceived knowledge, skills, and confidence in using VR. Nonetheless, actual uptake of VR in clinical routines remained limited. Enduring barriers included workflow misalignment, hierarchical decision-making structures, and the absence of a shared organizational vision and sustained leadership support. The longitudinal design revealed a dynamic pattern: early adoption hinged on individual capability and motivation, whereas maintenance depended on organizational opportunity and communicated support. These stage-specific shifts clarify why training alone does not translate into routine use and which organizational levers are most important when.

**Conclusions:**

VR training for therapists is a necessary but insufficient implementation strategy in mental health care. A longitudinal approach shows that successful implementation requires pairing training with organization-level changes that address opportunity barriers over time. By shifting from static evaluations of whether training works to a process-oriented focus on what support is needed at each stage of implementation, this study advances implementation science in digital mental health and offers actionable guidance for embedding VR in routine care.

## Introduction

### Adopting Virtual Reality in Mental Health Care

Virtual reality (VR) has emerged as an innovative technology to enhance treatment in mental health care. VR enhances exposure therapy and skills training, with potential benefits for outcomes, cost-effectiveness, and accessibility [1,2]. VR uses interactive, computer-generated environments to safely simulate real-life challenges tailored to individual needs [2]. The potential applications of VR technology in treating anxiety disorders, psychotic disorders, and aggression regulation disorders highlight its promise in enhancing treatment [3-7]. Yet, clinical adoption remains limited [8-11].

The implementation of VR in mental health care faces various challenges. Barriers that are often mentioned are the lack of knowledge and skills of therapists regarding the technical aspects of VR use and its integration into treatment [12-18]. In addition to these individual-level barriers, institutional obstacles such as limited resources, risk-averse organizational cultures, and a lack of governance or standardization frameworks frequently hinder the successful implementation of new technologies in health care settings [19]. Unlike traditional in-person therapy, VR requires both technical and therapeutic skills, such as conducting role-plays and integrating virtual tools into workflows [20]. For example, therapists not only need sufficient technical skills to use VR but also require different therapeutic and content-related skills, such as conducting a role-play in VR and integrating this new technology into their daily workflows [20]. To put these skills into practice, a significant behavior change is asked of these therapists. A qualitative study showed that despite their overall positive attitude toward VR and their intention to integrate it into their practices, they fall short in executing the necessary behaviors for successful integration [20]. This reflects an intention-behavior gap: therapists express intent but struggle to act accordingly [21].

One implementation strategy that can address these barriers and possibly bridge the intention-behavior gap is an extensive training program for therapists [20]. In health care services, training to integrate new treatment or technology in practice is often limited, creating a barrier to implementation [22]. When training does occur, it typically focuses on increasing the technical skills of practitioners to employ the technology in practice [23,24]. However, training is often not focused on therapeutic aspects of treatment, such as how to integrate the technology in treatment sessions, how to tailor VR to patients, or how to integrate it with therapeutic techniques. The introduction of VR, furthermore, seems to demand a shift in this approach, as it is not merely a technology. VR use fundamentally transforms how treatment is conceptualized and delivered; it requires therapists to adopt new roles, integrate virtual environments into treatment, and rethink traditional treatment structures [2,25]. Thus, training must address how VR reshapes therapy and what behavioral changes this requires from therapists.

### A Systematic Approach Toward Implementation

Implementation should not be an afterthought but rather be considered from the start of technology design, ensuring alignment with clinical needs and workflows [26]. Achieving this alignment requires more than individual effort—it depends on robust interorganizational collaboration and support structures that facilitate innovation at every stage of the implementation process [27]. To overcome common barriers, such as insufficient technical or therapeutic integration, tailored implementation strategies, like VR training, are essential [28]. In addition, it is important not only to identify useful implementation strategies and assess their effectiveness but also to understand why some strategies succeed while others fail [28]. It is important to consider determinants of behavior change in implementation. These determinants refer to the underlying factors that influence whether a person adopts or maintains a new behavior, such as their knowledge, beliefs, and external factors like social support [29]. By gaining insight into these factors, we can better tailor strategies to address the specific challenges therapists face. Ensuring they are aligned with the needs of the individuals involved and improving the chances of successful implementation [28,30].

**Figure 1.**
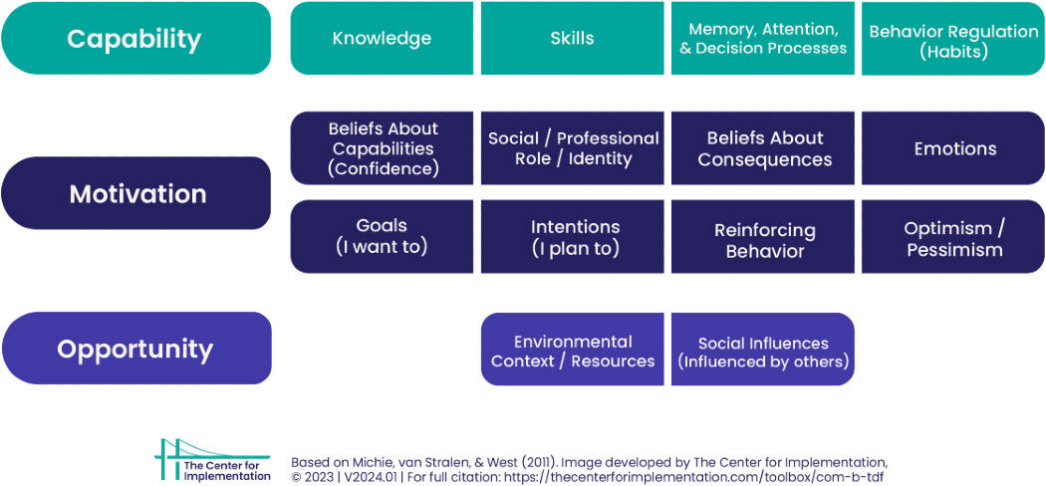
Capability, Opportunity, Motivation - Behavior & Theoretical Domains Framework. Adapted from Michie, S et al [34], reproduced with permission from The Center for Implementation [35], Version V2024.01.

This is where systematic mapping of determinants of implementation becomes important. To evaluate the implementation of VR in mental health care over time, it is recommended to map determinants that influence the implementation process, including therapist behavior, organizational factors, and other contextual elements [31]. The COM-B model frames behavior change in terms of capability, opportunity, and motivation and provides a structured approach to understanding what influences therapists’ implementation of VR technology [31,32]. This model is often complemented by the Theoretical Domains Framework (TDF), which then breaks these broad categories down into specific determinants, such as knowledge, skills, beliefs in consequences, and social influences [31]. Together, these models can provide both a high-level framework and a deeper understanding of the determinants that impact the implementation of VR [33]. Figure 1 illustrates the integration of both frameworks.

The 14 domains of the TDF framework are split up into the 3 categories of the COM-B (capability, opportunity, motivation – behavior) model. The capability category includes TDF domains related to cognitive and practical abilities required by therapists to use VR. These are essential for understanding how therapists can effectively integrate VR technology into their treatment practice. The motivation category includes TDF domains that reflect cognitive and emotional factors influencing motivation to engage in new behavior, such as using VR. The opportunity category includes the TDF domains that capture external factors that can either facilitate or hinder the implementation of VR in practice [33]. The COM-B and TDF complement each other in identifying implementation determinants to change behavior. Their combined use has been effective in various health care settings by systematically mapping influencing factors and tailoring implementation strategies accordingly [33,36].

These models are often used to assess the current situation by identifying barriers and facilitators. However, there is also potential to use them to evaluate implementation strategies. Both frameworks can help assess what changes occur as a result of a strategy, offering insight into which factors have been successfully addressed and which need further attention. The implementation strategy could be adapted accordingly. By mapping these factors in a longitudinal study design, a deeper understanding can be gained of how specific determinants contribute to the implementation of VR and how they change over time in response to the implementation strategies used [37]. Such insights could support the development of targeted, time-sensitive implementation strategies to enhance the effectiveness and sustainability of VR in mental health care practice.

### Evaluating the Impact of VR Training on Implementation in Mental Health Care

This study seeks to explore how an implementation strategy—a VR training program—impacts therapists’ VR use and their implementation behavior. This longitudinal qualitative evaluation aims to capture how this behavior evolves as therapists gain more experience with VR through the training program. The research questions guiding this study are:

1. How does a VR training program influence the determinants of VR implementation in mental health care, as defined by the TDF and COM-B model, from the perspective of therapists?
2. How do these determinants evolve over time, specifically between pretraining, posttraining, and follow-up, as therapists gain experience with VR through participation in the VR training program?

## Methods

### Study Design and Implementation Setting

This longitudinal study consisted of five phases: (1) a pretraining interview assessing expectations (T1), (2) a 6-session training over 3 months, (3) a posttraining interview assessing experiences (T2), (4) a 3-month implementation period, and (5) a follow-up interview evaluating VR use in practice (T3). The study took place within a large, multisite mental health care organization in the Netherlands that provides specialist care for patients with a broad range of psychiatric disorders, as well as developmental and forensic problems, across several outpatient locations and multidisciplinary teams. Five VR head-mounted displays were bought by the organization and were available during the study period. Devices were distributed across sites and could be booked by therapists; dedicated rooms were equipped for VR use. In addition, a mobile VR cart was available, allowing “one-button” start-up of a preassembled set to reduce setup time and technical burden. Device ownership and day-to-day management rested with the organization, while software maintenance and updates were provided by the vendor (CleVR). Technical support for troubleshooting was available via the CleVR helpdesk. Therapists could contact the research team by email for study-related questions or practical issues. Team management formally approved participation of their teams but was not actively involved in the VR training, day-to-day implementation decisions, or troubleshooting. Organizational support for VR was explicit during the study period: staff were offered a VR training program, and the organization financed the VR sets used. The manuscript follows the Standards for Reporting Implementation Studies (StaRI) guidelines to ensure comprehensive and transparent reporting of implementation research [38].

### Ethical Considerations

This study was approved by the Ethics Committee of the Faculty of Behavioural, Management and Social Sciences (BMS), University of Twente (request no. 231073). After being informed about the study and having the opportunity to ask questions, all participants provided written informed consent before the first interview. To protect participant privacy and confidentiality, audio recordings were transcribed and then deleted. Transcripts were anonymized and stored on an encrypted University of Twente drive, with access restricted to the research team. All data were handled in accordance with the General Data Protection Regulation. Participants received no compensation for participation, as the study was conducted as part of the VR training program they attended.

### VR Technology

**Figure 2.**
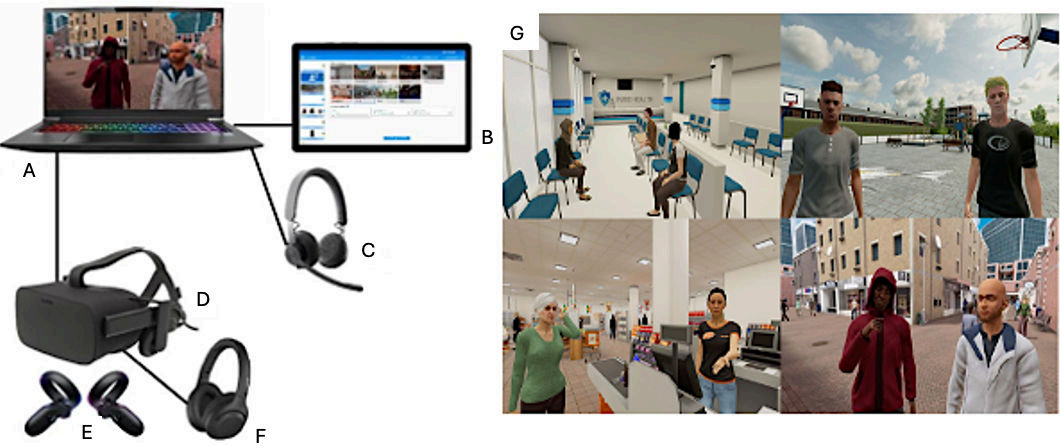
Setup of VR technology consisting of (A) a laptop; (B) a tablet with the dashboard; (C) a voice-morphing microphone; (D) a VR head-mounted display; (E) VR controllers; (F) headphones; (G) screenshots of virtual environments reproduced with permission from CleVR . VR: virtual reality. Copyright © CleVR [39].

In the VR training program, the interactive VR system “Social Worlds” of the Dutch company CleVR was used. This VR software makes it possible to bridge the gap between the real world and the treatment room by exposing patients to a variety of realistic virtual environments and letting them interact with a broad range of virtual characters. The patient can navigate multiple environments, including a supermarket, a shopping street, or a home environment. Furthermore, the patient can engage in role-play with virtual characters, in which these characters are embodied by the therapist using a voice-morphing microphone. The therapist has control over the character’s voice, movements, facial expressions, and body language through a user dashboard. The dashboard allows therapists to dynamically control virtual environments and character behavior. The setup of the VR technology is illustrated in Figure 2. This technology enables the development of customized VR scenarios tailored to diverse client needs and treatment goals.

### Implementation Strategy: VR Training Program

Initially, the implementation process of the VR technology described in this paper started in 2019, with the decision of management to buy two VR sets of CleVR. The current training builds on lessons from an earlier pilot [20]. The VR training program that is described in this study is a new implementation strategy that was put into practice based on the lessons learned from that previous pilot [20]. To develop a training that has a solid foundation to help integrate VR in practice, a systematic approach was used to address hindering and facilitating aspects related to the implementation of VR found in previous studies [11,20]. These implementation factors identified in previous studies were categorized according to the domains of the Consolidated Framework for Implementation Research (CFIR) [40], which provides a broad perspective on implementation at multiple levels (eg, inner setting, individuals, and intervention characteristics). In contrast, this study applies the TDF and COM-B model to specifically examine how individual therapists’ behaviors evolve during VR implementation. This distinction allows us to build on prior findings while focusing on the mechanisms underlying therapist behavior change.

The training program was developed in a cocreation process with a multidisciplinary team consisting of 2 researchers, 2 policy officers, and 1 therapist. The translation of implementation factors into concrete training activities was done through an iterative process, in which the team ensured that each barrier was addressed through targeted exercises, discussions, or hands-on practice. Additionally, the training design was informed by existing structured training programs for other treatments, such as cognitive behavioral therapy and eye movement desensitization and reprocessing, which emphasize the importance of thorough education, supervision, and skill development before clinical application [41,42]. The main goal of the VR training program was to equip participating therapists with the knowledge and skills necessary to proficiently use VR in therapeutic practice. The 6-session program covered technical setup, therapeutic integration, and reflective practice through role-plays, peer feedback, and supervised application in clinical sessions. The training was delivered by 2 trainers: a policy officer, who ensured the right organizational conditions and provided technical support, and a therapist, who guided participating therapists in the clinical application of VR.

### Participants

Interviews were conducted with 11 therapists who all participated in the VR training. Inclusion criteria for therapists were that they (1) currently worked for the mental health care organization, (2) were involved in any type of mental health care treatment with inpatients or outpatients, and (3) participated in the VR training program.

The recruitment of therapists was carried out by the coordinator and trainer of the VR training program, an innovation policy officer. All employees who met the inclusion criteria were invited via the internal portal or by their manager. Interested therapists registered via email. Of 12 therapists participating in the training, 11 participated in the study (Table 1). Of those, 7 completed the training and all three interviews, and 4 dropped out due to illness, scheduling conflicts, or job change.

**Table 1.** Characteristics of participating therapists.

Participant ID	Function	Work experience in mental health care (in years)	Patient group	Participated in interview round
1	Psychologist in training to become behavioral therapist	6	Complex trauma	1, 2, 3
2	Cognitive behavioral therapist and system therapist	19	Forensic	1, 2, 3
3	Psychologist	4	Anxiety, mood, and personality disorders	1, 2, 3
4	Social psychiatric nurse	24	Bipolar and psychotic disorders	1, 2, 3
5	Social pedagogical counselor	6	Anxiety, mood, and personality disorders	1, 2, 3
6	Psychomotor therapist	7	Complex trauma and anxiety and mood disorders	1, 2, 3
7	Social worker and social psychiatric nurse	1	Youth	1
8	Psychologist	2	General	1, 2
9	Clinical psychologist and cognitive behavioral therapist	20	Youth	1
10	Nurse practitioner and lead practitioner	33	Addiction	1
11	Social worker	23	Youth	1, 2, 3

### Materials and Procedure

Data were collected by one researcher (MMTEK) through in-depth, semistructured interviews. Three interview schemes were developed to gain in-depth insights into the expectations and experiences of the therapists with the VR training program. The first draft of the interview protocols was improved based on a pilot test with a therapist—who did not take part in the VR training—and in consultation with another researcher (HK).

After the therapists were informed about the study, they had the opportunity to ask questions. They signed the informed consent form before the first interview took place.

The first interview took place in October 2023. Therapists were asked about their expectations regarding the VR training program, focusing on activities, possible barriers, and advantages of the program. Lastly, they were asked to formulate concrete personal goals regarding their VR usage over the next 6 months.

The second interview took place in January 2024, directly after the training ended. The therapists were asked about their experiences with the VR training program. Questions were asked about the match between their expectations before the training and their experiences after. Finally, it was discussed to what extent therapists achieved their personal goals for VR use.

The third follow-up interview took place in April 2024, three months after the VR training ended. Mainly, the added value of VR and additional points of improvement in the implementation process of VR technology were discussed. Finally, they were asked about expectations for future VR use in practice.

All interviews took place online, via Microsoft Teams, a videoconferencing program. They were conducted by one researcher (MMTEK) and were audio recorded. On average, the interviews took 27 minutes, ranging from 15 to 37 minutes. The interview protocols are presented in Multimedia Appendix 1.

### Data Analysis

Audio recordings of the interviews were transcribed verbatim by one researcher (MMTEK). All transcripts were read to become familiar with the data and identify meaningful fragments in relation to the COM-B categories and the TDF domains. Data were analyzed using theoretical thematic analysis [43,44]. In this approach, themes are guided by theory and study aims, and coding links the data to those themes [43]. In this study, this deductive, theory-led approach was deliberately selected as a first step to evaluate implementation determinants and to examine how positive and negative evaluations within each TDF domain changed over time.

The first version of the deductive coding scheme was developed by one researcher (MMTEK). Another researcher (HK) remained consistently involved in the process, providing continuous feedback and oversight on theme definitions and preliminary interpretations. Fragments were first categorized deductively based on the categories of the COM-B model and the domains of the TDF. Second, fragments per domain were categorized as positive or negative to identify key strengths and areas for improvement. Negative evaluations were analyzed alongside suggested improvements, providing actionable insights for effective implementation. Mixed statements were split into positive and negative components when conceptually distinguishable.

In the analysis, we paid attention to how these determinants evolved across the 3 time points to observe shifts in capability, motivation, and opportunity over time as therapists gained more experience with VR. We looked at both what changed qualitatively (the content of therapists’ accounts) and how often it occurred. First, we compared the qualitative content of coded fragments across time to identify shifts in how capability, motivation, and opportunity were described, using exemplar quotes to illustrate typical and divergent trajectories. Second, we counted, for each TDF domain and timepoint, the number of fragments labeled as positive or negative and visualized these as paired bar charts to support pattern recognition. The unit of analysis was meaningful fragments. Each fragment was assigned to one best-fitting TDF domain to avoid double-counting. In case of doubt, we assigned the fragment to the domain that best captured its dominant meaning, refining decision rules through discussion within the research team. In line with current guidelines on theoretical and reflexive thematic analysis, oriented to prevent quantifying qualitative results, meaning that numbers were only used descriptively [43,44].

Ten percent of transcripts were double-coded independently by a second researcher (LAMK), yielding 84% agreement before consensus discussion. Discrepancies were resolved collaboratively and led to minor refinements to the codebook, which were applied consistently. Given the theory-driven coding scheme and meaningful units of analysis, percentage agreement plus consensus discussion was deemed an appropriate way to assess convergence between coders [44,45].

Trustworthiness and transparency were supported by an audit trail including a documented Microsoft Excel codebook with versioned updates after consensus decisions, periodic peer debriefing with collaborating researchers, and brief analytic (reflexive) memos to record key decisions and underlying reasoning [46]. Interviews and initial coding were conducted by the first author (MMTEK), a PhD candidate/researcher with a background in health psychology and implementation science and prior experience with VR implementation in mental health care. MMTEK contributed to the development of the VR training in a research capacity but was not involved in its commercial development or delivery (eg, as developer, implementation partner, or trainer) and reported no conflicts of interest. Participating therapists were recruited across locations and did not know MMTEK. At the start of interviews, participants were explicitly encouraged to provide open and critical feedback. Reflexive discussions during analysis involved HK (expertise in eHealth development, implementation, and evaluation) and LAMK (master's student on VR implementation) to reflect on interpretations and refine the codebook where needed.

## Results

### Overview of Findings

In this section, the findings of this study on determinants influencing the implementation of VR in mental health care are presented. The findings from each interview round are presented as tables in the following subsections, corresponding to the pretraining, posttraining, and follow-up interviews, respectively. The determinants are derived from the combined COM-B model [34] and the TDF [31], as illustrated in Figure 1. The evolution of determinants over time is presented as figures in the following subsections.

### Capability

**Figure 3.**
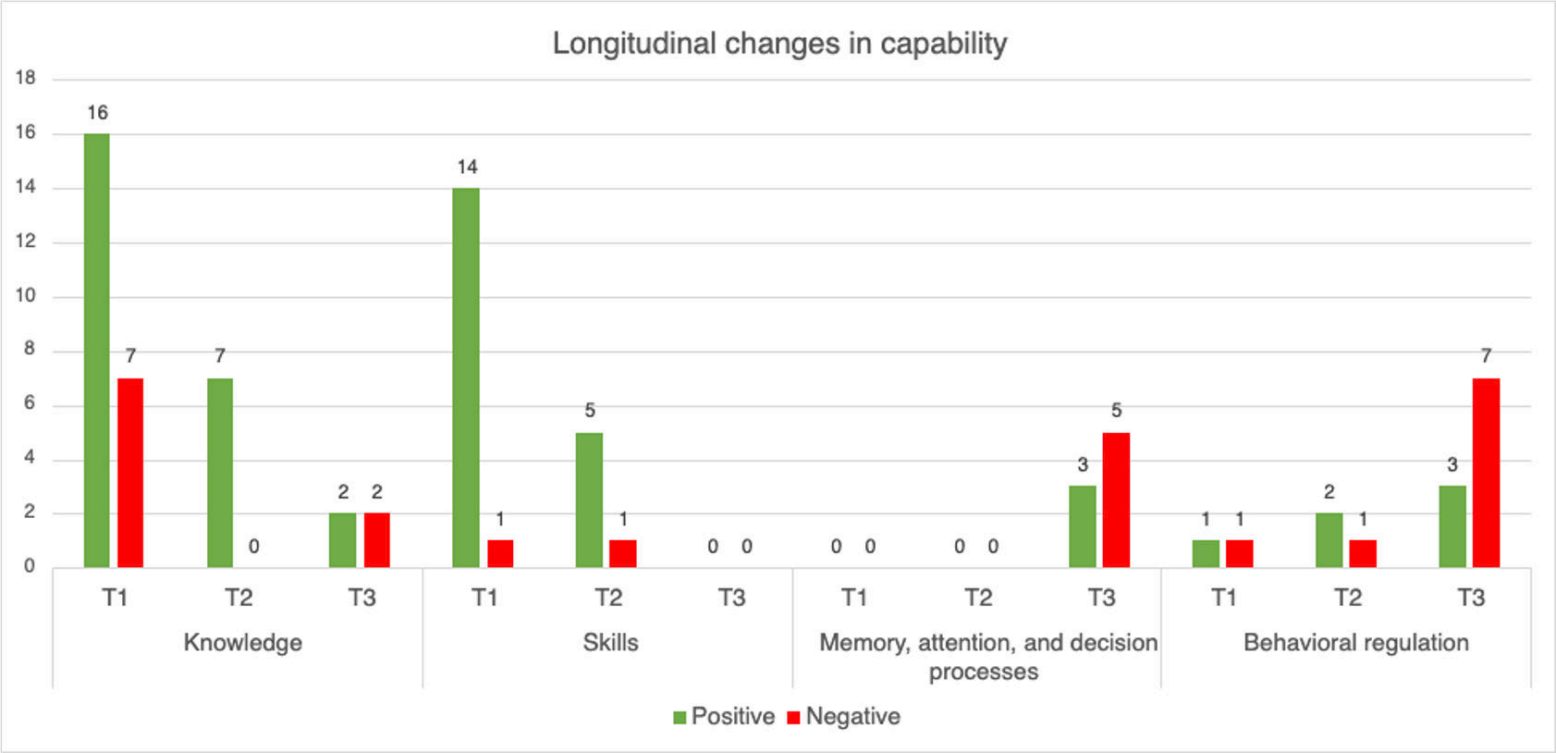
Positive and negative evaluations per timepoint (T1-T3) for capability subdomains (counts of coded fragments). T1 refers to the pretraining interview, T2 to the immediate posttraining interview, and T3 to the follow-up interview 3 months after training completion.

The *capability* category explores determinants that relate to the therapists’ psychological and physical capacity to implement VR in their treatment practice [34]. In Table 2, all determinants within the capability category that influence the implementation of VR technology in mental health care, according to therapists, are described. Figure 3 illustrates how these determinants evolved over time.

**Table 2.** Determinants related to capability.

Domain, definition, and interview round	N^a^_tot_	N+^b^	N–^c^	Quote+	Quote–
Knowledge					
Therapists’ awareness and familiarity with using VR^d^					
Round 1	23	16	7	“I know there are colleagues who haven’t had such extensive training…I don’t think that’s okay because I believe you need to have sufficient knowledge before using it.” [Participant 2]	“Yes, of course, you need to know how it [VR] works, but right now I have no idea how to go about it.” [Participant 4]
Round 2	7	7	0	“I appreciated that some background information was shared. I usually prefer practicing hands-on, but it’s definitely good to know the theory behind it as well, and you can certainly find that in the training.” [Participant 6]	Not mentioned
Round 3	4	2	2	“I do have the knowledge to use it. If I were to start doing it now, it would come back to me right away.” [Participant 6]	“I notice that I still sometimes find it difficult to know when it’s appropriate to use it or not…But I think it’s simply a matter of gaining experience.” [Participant 5]
Skills					
Therapists’ ability or proficiency in VR use acquired through practice					
Round 1	15	14	1	“I expect to become skilled in operating the equipment, to learn about its possibilities and applications in treatment, and to gain insight into suitable case studies.” [Participant 11]	“At the moment, I don’t feel well-prepared to use VR; I think I still need to get comfortable with the technical aspects.” [Participant 2]
Round 2	6	5	1	“I do think I could use VR in practice now. I believe I can do it.” [Participant 3]	“I didn’t learn how to apply VR in a group setting. That part remained really vague in the training. How do you actually do that in practice?” [Participant 5]
Round 3	0	0	0	Not mentioned	Not mentioned
Memory, attention, and decision processes					
The extent to which using VR is part of therapists’ regular practice					
Round 1	0	0	0	Not mentioned	Not mentioned
Round 2	0	0	0	Not mentioned	Not mentioned
Round 3	8	3	5	“I do think about VR as a treatment option when I start working with someone. During the introduction, I consider whether it would be a good fit or not.” [Participant 4]	“It’s still not an automatic part of my thinking when I assess someone. I’m just not focused on it at that moment. It’s not part of my automatic response.” [Participant 3]
Behavior regulation					
Therapists’ ability to self-monitor and action plan to use VR					
Round 1	2	1	1	“Yes, I would appreciate having peer supervision groups and time with colleagues. Also because I know that I wouldn’t keep this in focus on my own. So I find that very beneficial.” [Participant 5]	“If I start using VR, it requires quite a bit of planning and organization.” [Participant 1]
Round 2	3	2	1	“My learning style is that I like to have everything clear in advance…Some people are more like, ‘I’ll just do it and see what happens.’ I’m definitely more on the other side.” [Participant 8]	“Often, you plan in this work so shortly in advance, and then you don’t have much time beforehand to consider using VR.” [Participant 2]
Round 3	10	3	7	“I do try to practice as much as I can on my own. I regularly make an effort to sit down with it so I can keep my knowledge up to date on how to use it. Otherwise, it will be even harder when I need to use it with patients.” [Participant 5]	“The reason I use VR less now is that you really need to have a clear plan in advance and reserve the equipment. So all those preparations don’t really align with my current flexible working style.” [Participant 6]

a The number of times the determinant is mentioned in that specific interview round.

b The number of times the determinant is mentioned as a positive point in that interview round.

c The number of times the determinant is mentioned as a point of improvement in that interview round.

d VR: virtual reality.

In total, *knowledge* was the most frequently mentioned determinant across all interview rounds, with positive remarks about the extensive training program increasing their knowledge, but also negative remarks about the overall difficulty of applying VR in treatment. A noteworthy shift was observed in therapists’ perceptions regarding their knowledge related to VR use. Initially, therapists highlighted their lack of knowledge of VR, particularly in its application, indications, contraindications, and integration into treatment. During the training, therapists gained knowledge, and 3 months after the training concluded, all therapists mentioned retained knowledge of VR usage in treatment. Some therapists mentioned needing more practice to apply the knowledge in practice. All therapists indicated that the training had addressed initial gaps, fostering confidence in their ability to use VR in clinical practice.

Therapists mentioned that their *skills* related to VR use also improved. Therapists expressed a high level of proficiency in using VR and integrating it into their treatment after the training, with few negative comments on their competence. This positive shift suggests that the therapists felt they had acquired the necessary skills to incorporate VR into their practice. Therapists mentioned that they retained their VR skills over time. Interestingly, in later interviews, therapists hardly mentioned skills as a topic for discussion. While initially perceived as a key learning objective, the focus on skill development diminished after the training. At the same time, other factors received more attention as the interviews progressed, indicating that other determinants became more pressing.

Regarding *memory, attention, and decision processes* and *behavioral regulation*, therapists acknowledged 3 months after the training that VR had not yet become a standard treatment option. Despite their increased confidence in using VR, they did not automatically think of it as a standard option when creating their treatment plans. Many therapists still reverted to more familiar treatment methods, highlighting the lack of automatic integration of VR into their decision-making processes. The structured planning and coordination for VR use was mentioned as a challenge, as therapists’ treatment routines typically involved spontaneous adaptations based on patient needs, rather than the scheduling that VR requires in practice. Interestingly, this topic had not been discussed in the first two interviews, possibly indicating that it had not been a priority in their initial thinking.

Overall, therapists felt confident in their capacity to use and integrate VR into their clinical practice, indicating the retention of knowledge and skills over time. Despite this capability, therapists mentioned that they did not use VR as often as they would have thought when they started the training.

### Motivation

**Figure 4.**
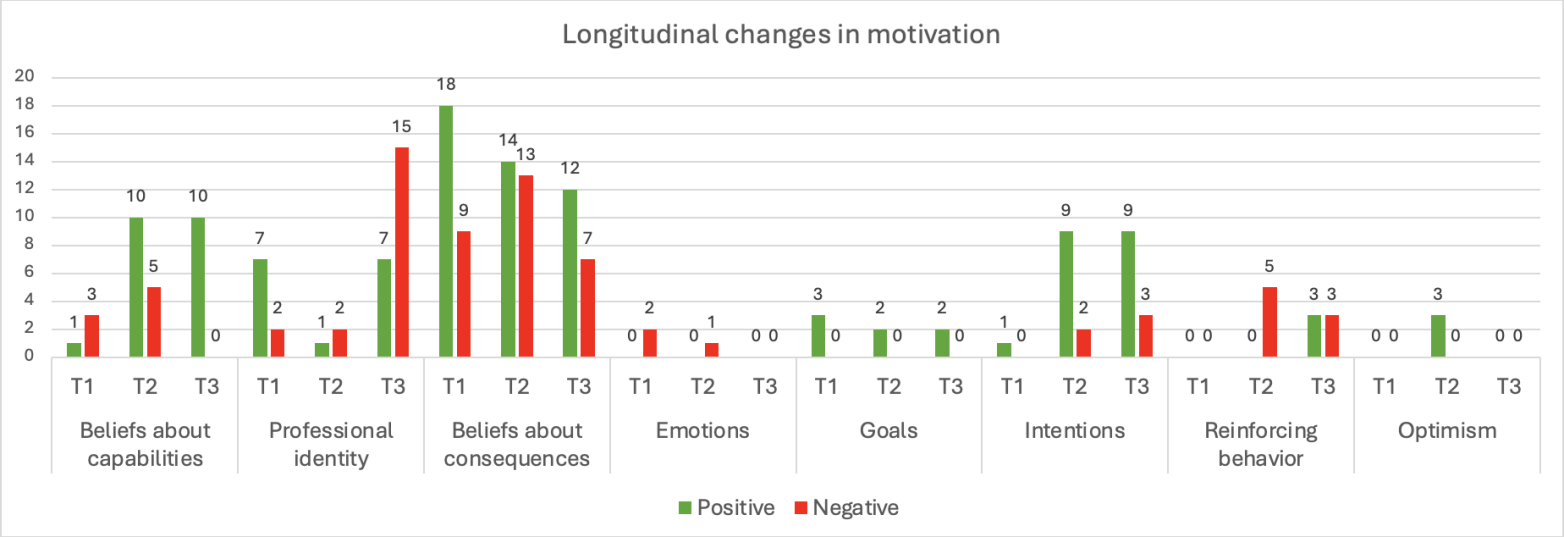
Positive and negative evaluations per timepoint (T1-T3) for motivation subdomains (counts of coded fragments). T1 refers to the pretraining interview, T2 to the immediate posttraining interview, and T3 to the follow-up interview 3 months after training completion.

The *motivation* category explores determinants that relate to therapists’ internal process that initiates, directs, and sustains goal-oriented behavior. It includes not just goals and intentions, but also habitual processes and emotional responses [34]. In Table 3, all determinants that played a role in shaping therapists’ motivation to implement VR into mental health care are listed. Figure 4 illustrates how these determinants evolved over time.

**Table 3.** Determinants related to motivation.

Domain, definition, and interview round	N^a^_tot_	N+^b^	N–^c^	Quote+	Quote–
Beliefs about capabilities					
The therapists’ confidence in their ability to implement VR^d^					
Round 1	4	1	3	“You don’t have to get it all perfect right away. You can take the patient along and explore it together a bit. I think that’s very valuable as well.” [Participant 8]	“I approach it quite blankly, and I’m not sure if I can actually do it.” [Participant 5]
Round 2	15	10	5	“Yes, things did go wrong sometimes. For example, the batteries ran out, or I had to calibrate the equipment, but that’s something you’ll encounter in daily practice as well. So, I’ve already dealt with it. I don’t panic anymore when something doesn’t work because I know I can fix it myself. I understand how it works—it’s just part of the process.” [Participant 4]	“It will take some time before it feels comfortable in a treatment setting. That sense of ease needs to grow as you do it more often.” [Participant 2]
Round 3	10	10	0	“I think the training has definitely been beneficial in terms of practice, and that’s made it clearer in my mind now. I’m also capable to use it.” [Participant 6]	Not mentioned
Professional identity					
The extent that implementation of VR is perceived as part of the therapists’ role and their view on mental health care					
Round 1	9	7	2	“Look, we also need to keep up with our future. I believe that’s a positive development.” [Participant 8]	“I’m also very curious about what my role as a therapist will be exactly. Should I just observe or coach? I’m not sure about that yet.” [Participant 3]
Round 2	3	1	2	“[The use of VR] is really about discipline and time management. I believe that ownership comes from within yourself.” [Participant 11]	“It’s always the case in mental health care that developments progress slowly.” [Participant 1]
Round 3	22	7	15	“It wouldn’t surprise me if VR becomes a standard component of mental health care in the Netherlands. That this option is available everywhere.” [Participant 3]	“I think we all act as if that hierarchy doesn’t exist and that everyone can share ideas, but it is definitely there. Emotionally, that freedom isn’t really present. Someone else determines the policy, what I do. I don’t do that myself.” [Participant 3]
Beliefs about consequences					
The therapists’ beliefs about the advantages or disadvantages of using VR					
Round 1	27	18	9	“In my view, it will especially help with exposure. It’s an added value because you can create a setting that you wouldn’t normally be able to create in your treatment room.” [Participant 3]	“I find it interesting, but I’m not completely convinced of its value yet. I’m still exploring it.” [Participant 2]
Round 2	27	14	13	“I see it as an enrichment for our team. It’s much more effective. You can practice multiple situations with someone. For example, a patient with distrust was able to practice various scenarios, and he was completely surprised by what he could already do. And then it’s also easier to do it for real.” [Participant 4]	“I actually thought there were many more possibilities in VR in terms of situations, but that doesn’t seem to be the case.” [Participant 5]
Round 3	19	12	7	“I believe that VR definitely adds value.” [Participant 1]	“I’m managing just fine now, and it’s not an added value compared to the resources that already exist.” [Participant 11]
Emotions					
Therapists’ complex response pattern when dealing with VR, involving cognitive, affective, and physiological elements					
Round 1	2	0	2	Not mentioned	“Yes, I do have a healthy dose of anxiety [before the training], but that’s something I can overcome.” [Participant 5]
Round 2	1	0	1	Not mentioned	“But there were always technical issues, and I found that really annoying...I can’t help but feel disappointed with the technical side of it.” [Participant 3]
Round 3	0	0	0	Not mentioned	Not mentioned
Goals					
Therapists’ mental representation of outcomes or end states that they want to achieve in relation to VR use					
Round 1	3	3	0	“It’s important to have some kind of focal point or goal in advance...That way, you feel a sense of responsibility and prepare for it.” [Participant 8]	Not mentioned
Round 2	2	2	0	“I’ve really learned those principles now. It also takes time to start trying and further exploring them. But that goal has definitely been achieved.” [Participant 6]	Not mentioned
Round 3	2	2	0	“The goal is really to start using it more. I hope that will be possible.” [Participant 4]	Not mentioned
Intentions					
A conscious decision to implement VR					
Round 1	1	1	0	“I saw on the intranet that there would be VR training, and it had already interested me for some time. So when I saw this, I thought, ‘It seems really fun to me to do that as a therapist.’ ” [Participant 8]	Not mentioned
Round 2	11	9	2	“After this training, I’m really ready to go. I now have a patient with whom I’ve used VR, and now we can explore how we can shape that [further VR treatment].” [Participant 2]	“There is no motivation here [within my team] to roll it out on a large scale and bring it to attention, simply because we say: this is not what we expected or what we need.” [Participant 11]
Round 3	12	9	3	“Yes, I definitely intend to use VR in the future.” [Participant 4]	“I don’t have the intention to use it right now. At the moment, I don’t feel obligated to use it.” [Participant 3]
Reinforcing behavior					
The extent of recognition and reward the therapists expect to receive when implementing VR					
Round 1	0	0	0	Not mentioned	Not mentioned
Round 2	5	0	5	Not mentioned	“It’s actually childish that it’s necessary, but I would really make practicing mandatory. With the busyness of the week, you quickly say, ‘Oh, I’ll do that next week.’ ” [Participant 1]
Round 3	6	3	3	“It’s already great that there is an opportunity to take a training course, so I don’t expect anything specific in return. A certificate or something would be nice, though.” [Participant 6]	“I actually consider a certificate to be the minimum. It would be nice to have something to demonstrate for yourself and for your CV that you’ve been trained in this and that you’ve invested in it.” [Participant 5]
Optimism					
Therapists’ confidence that the implementation of VR will be attained					
Round 1	0	0	0	Not mentioned	Not mentioned
Round 2	3	3	0	“Yes, I really hope the VR set stays here. It is definitely achievable, but there needs to be more focus on it. Success stories are always motivating, right?” [Participant 4]	Not mentioned
Round 3	0	0	0	Not mentioned	Not mentioned

a The number of times the determinant is mentioned in that specific interview round.

b The number of times the determinant is mentioned as a positive point in that interview round.

c The number of times the determinant is mentioned as a point of improvement in that interview round.

d VR: virtual reality.

At the outset, many therapists expressed reservations in their *beliefs about capability* to integrate VR into their practice. However, throughout the training, confidence grew, and all therapists reported a belief in their capability to use VR effectively. The determinant *beliefs about consequences* of VR use emerged as a key factor, with therapists recognizing the potential of VR for exposure therapy and appreciating its benefits, such as providing a controllable and safe environment for various scenarios. A therapist even suggested that VR could be more effective than traditional treatments, especially in helping patients transfer coping strategies to real-life situations. Despite these positive beliefs, concerns remained about the lack of realism in some virtual scenarios and the challenges in finding suitable patients for VR treatment. Most of their patients were able to follow exposure therapy outside the clinic, making the use of VR unnecessary. Other patients required medication for more pressing symptoms before engaging in VR treatment.

The *professional identity* of therapists became more prominent as the training progressed. Therapists increasingly saw the potential for VR to become a standard treatment option in mental health care. However, some reported feeling constrained by hierarchical team dynamics that hindered their willingness to stimulate VR use after the training concluded. The perception of limited autonomy within their teams restricted their motivation to advocate for the continued use of VR.

Furthermore, the therapists’ attention to *reinforcing behavior* regarding the use of VR increased over time. Most therapists advocated for stricter requirements for participating in VR training and receiving certification to encourage responsible VR use. They believed that setting higher entry requirements would enhance motivation, accountability, and discipline among those wishing to integrate it into their therapeutic practice.

In the interviews, emotions, goals, intentions, and optimism were mentioned less frequently. Relating to *emotions*, therapists mentioned some anxiety about using VR, particularly about technical issues that may occur, but these emotions decreased over time. Regarding *goals*, therapists highlighted the importance of having a clear objective when using VR, with some aiming to integrate it more into their practice. *Intentions* were expressed mostly positively, especially after training, with therapists feeling ready to implement VR, though some struggled with team-wide motivation. Lastly, *optimism* for VR’s future in treatment was expressed, but this was less frequently discussed.

### Opportunity

**Figure 5.**
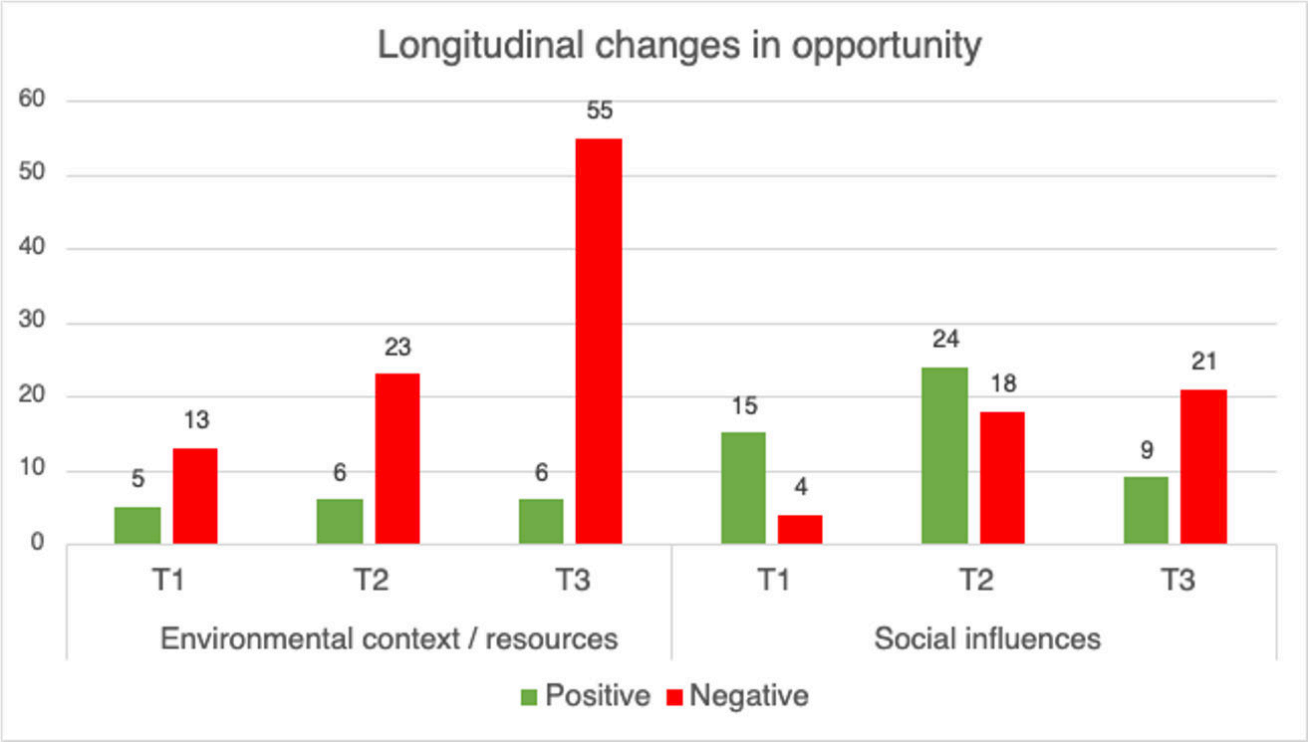
Positive and negative evaluations per timepoint (T1-T3) for opportunity subdomains (counts of coded fragments). T1 refers to the pretraining interview, T2 to the immediate posttraining interview, and T3 to the follow-up interview 3 months after training completion.

The *opportunity* category explores determinants that relate to all the factors that lie outside the therapist that make the implementation of VR possible or hinder it [34]. In Table 4, both determinants within the opportunity category are presented. Figure 5 illustrates how these determinants evolve over time.

**Table 4.** Determinants related to opportunity.

Domain, definition, and interview round	N^a^_tot_	N+^b^	N–^c^	Quote+	Quote–
Environmental context/resources					
Any circumstance of a therapist’s environment that encourages or discourages VR^d^ implementation					
Round 1	18	5	13	“Well, we have a device available at the location, so it’s quite convenient. My supervisor has also asked me if I would like to do this training, and the fact that I’m going to take this training gives me the impression that I’m being supported by management.” [Participant 2]	“Well, the availability of the set is important, of course. Not having it at this location does create a barrier.” [Participant 4]
Round 2	29	6	23	“Actually, the training was really accessible for me because it was on my workday and at the same location. I could just go down the hall.” [Participant 11]	“You often run into technical problems, so you have to solve those first, along with setting up the system. In the end, a lot of time is simply lost.” [Participant 5]
Round 3	61	6	55	“Yes, there are plenty of VR sets available, and I know colleagues I could reach out to if there’s an issue or where I can call for assistance. That part is well taken care of.” [Participant 5]	“I don’t get the impression that the organization has a very clear vision regarding VR. It is mentioned in the course, but there’s little information on where the organization stands.” [Participant 6]
Social influences					
Interpersonal processes that can cause therapists to change their thoughts, feelings, or behaviors					
Round 1	19	15	4	“The advantage of peer supervision is that if you encounter something, you can discuss it right away and make adjustments immediately. That’s valuable because it helps keep the attention on VR, right?” [Participant 4]	“The motivation or encouragement from management is very limited. I discovered this training on my own initiative based on my professional development and personal interest.” [Participant 10]
Round 2	42	24	18	“Yes, it’s more ‘alive’ than I expected. I also come across other people who are using VR.” [Participant 3]	“Yes, the idea was that there would be peer supervision, but that isn’t happening here right now. So I notice that I’m somewhat searching for where to turn if there’s an issue and where I would end up.” [Participant 2]
Round 3	30	9	21	“We do have a chat in Teams where we can ask each other questions within the training group. If I were to post a message saying, ‘Hey, I’m stuck on this,’ I know I would get a quick response.” [Participant 1]	“You can tell that it’s not always well-known within the treatment team. You really have to work hard to raise awareness about it in the team and share some information.” [Participant 5]

a The number of times the determinant is mentioned in that specific interview round.

b The number of times the determinant is mentioned as a positive point in that interview round.

c The number of times the determinant is mentioned as a point of improvement in that interview round.

d VR: virtual reality.

Both determinants, *environmental context/resources* and *social influences*, emerged as critical in understanding therapists’ experiences and attitudes toward the implementation of VR. Initially, therapists identified the availability of VR sets and training as facilitating factors. However, as the study progressed, these were overshadowed by a growing sense of frustration due to an increasing number of challenges, such as technical difficulties with the VR equipment and issues with reserving the necessary resources. Therapists reported that these complications, combined with overwhelming workloads, led to an increasing sense of dissatisfaction and left little room to explore and integrate VR into their treatment practices.

A significant lack of organizational vision and sustained support after the training regarding using VR in treatment was also noted. Therapists felt that management did not prioritize VR and provided limited support or communication after the training ended. This clear lack of direction from the organization created an environment where therapists felt largely unsupported in their attempts to integrate VR into their treatments. By the final round of interviews, many therapists voiced the need for sustained organizational support and a robust vision for integrating VR and innovations in general into mental health care.

Regarding *social influences*, therapists initially experienced a sense of enthusiasm and community among colleagues, but this supportive atmosphere diminished over time. Therapists reported feelings of isolation and disappointment stemming from a lack of ongoing encouragement from colleagues, team leaders, and management. Many expressed that the absence of engagement and motivation from leadership further exacerbated these feelings, decreasing their drive to use VR. Three months after the training concluded, therapists indicated a need for a more active role from management and for increased awareness and advocacy within their teams to foster a supportive environment.

Overall, these two determinants were more frequently discussed than other determinants in the TDF framework. Issues ranged from broader organizational challenges to specific team dynamics and individual colleague interactions.

### Synthesis of Longitudinal Transitions and COM-B Interactions

**Figure 6.**
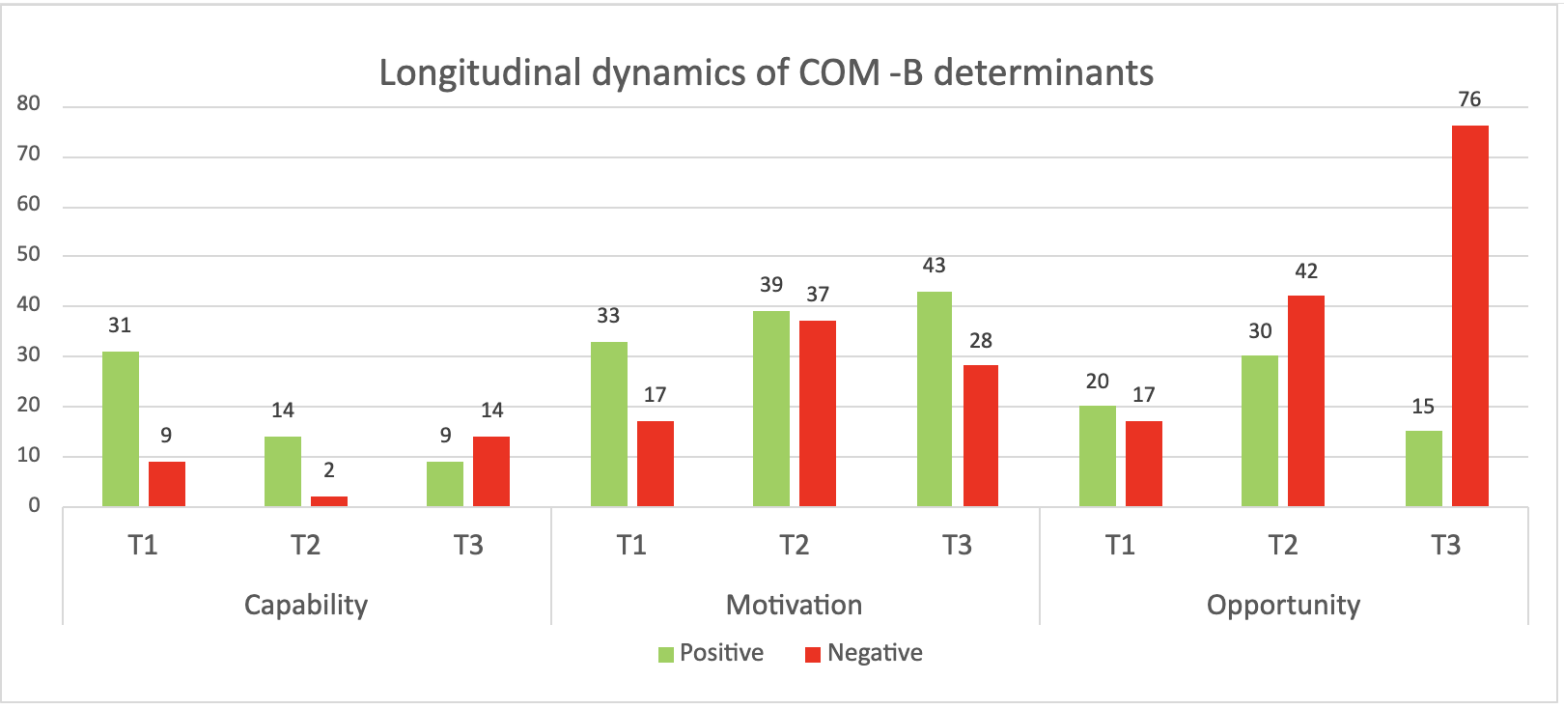
Positive and negative evaluations per timepoint (T1-T3) across COM-B categories (counts of coded fragments). T1 refers to the pretraining interview, T2 to the immediate posttraining interview, and T3 to the follow-up interview 3 months after training completion. COM-B: capability, opportunity, motivation – behavior model.

Across interviews, patterns differed by COM-B category (Figure 6). Capability-related barriers, such as lack of knowledge and skills, decreased during training, while positive evaluations related to capability became less prominent in the immediate posttraining interviews. This does not reflect loss of skill or knowledge, but rather a shift in focus: after training, capability felt largely established in therapists and was therefore less frequently discussed. At the third follow-up interview, capability negatives increased, driven mainly by memory, attention, and decision processes and behavior regulation. Here, therapists reported forgetting to use VR in the flow of daily care or struggling to embed it into routines, rather than lacking knowledge or technical skill.

Motivation remained overall positive and strengthened over time. Critical appraisals peaked immediately after training, reflecting a more realistic weighing of VR’s value against daily practice demands. By follow-up, negative motivational evaluations decreased, consistent with consolidation among the therapists who found workable ways to apply VR and experienced its added value in day-to-day care.

In the descriptive counts, opportunity factors emerged as the most persistent bottleneck for sustained use. Positives rose during the training but halved at follow-up, while negative evaluations related to opportunity increased sharply. These shifts were primarily attributed to organizational conditions. Recurrent themes included the absence of a clear organizational vision and plan for VR, lack of visible leadership support, and limited availability of protected time. In short, once therapists became sufficiently capable and remained motivated, opportunity factors hindered translation into routine care.

While no firm conclusions can be drawn based on the quantification of qualitative data, the findings suggest an interaction between individual and organizational factors: training helps build capability, and once therapists feel able and willing to use VR, gaps in organizational vision, resources, and support become more salient. In our data, VR use remained sporadic when these organizational conditions were not in place, even when therapists reported sufficient motivation and skills.

## Discussion

### Principal Results

This longitudinal evaluation explored how a specific implementation strategy—a VR training program—impacts therapists’ VR use and implementation behavior. The evaluated behavior determinants are derived from the TDF and COM-B model [34]. The key findings are related to the capability, motivation, and opportunity of therapists to implement VR. These findings suggest an ongoing, interconnected process rather than 3 isolated measurement timepoints. Capability was established through the training. Therapists generally felt they were able to use VR. The later rise in negative evaluations related to capability at follow-up did not signal loss of skill, but rather the practical demands of remembering to use VR in busy daily workflows and embedding it into routines. This signaled issues of memory, attention, and behavior regulation rather than competence. Motivation to use VR stayed broadly positive. Right after training, therapists briefly weighed VR’s value against day-to-day pressures. Where VR proved to be of added value in real cases, motivation stabilized. Posttraining, the sporadic use observed across therapists could be explained by organizational conditions: without a clear managerial vision, visible leadership engagement, protected time, and reliable technical support, intention and capability did not translate into routine use. Importantly, capability gains did not remove obstacles; they made the organizational layer more visible. This underscores that implementation is layered rather than strictly sequential. Strategies should therefore act across different levels. For example, aligning with all the different CFIR domains.

### Comparisons to Prior Work and Implications for Practice

The VR training program effectively supported knowledge acquisition and positively impacted various other determinants, including the development of skills necessary for VR use in practice. Therapists reported increased confidence in both technical and therapeutic applications. These findings align with research emphasizing that successful implementation of new technologies requires more than technical training; professional development should focus on broader topics such as privacy, ethical and legal considerations, clinical competencies, and administrative tasks [47]. However, despite these self-reported improvements, VR was not consistently integrated into daily treatment practice after the training concluded. Therapists tended to revert to familiar treatment methods rather than incorporate VR as a standard part of their treatment. This is often seen in VR implementation, as therapists tend to feel more comfortable and confident in the effectiveness of their familiar methods, and these methods are perceived as more straightforward and reliable [48-50]. Some other key barriers to implementation were the high perceived workload and time constraints, which made it difficult for therapists to reserve and set up VR equipment in time for treatment. This highlights the importance of considering context-related obstacles in implementation efforts. Similar barriers were reported in a Belgian eHealth implementation study, which stated that high workload and lack of time often hinder the adoption of new technologies and innovations in mental health care [51].

Regarding motivation to use VR, therapists generally viewed VR as a valuable tool, particularly for interventions such as exposure therapy, where it could lower the threshold for real-life exposure. This positive attitude toward the technology reflects growing recognition of VR’s potential to enhance treatment. However, despite this enthusiasm, concerns about the perceived consequences of using VR in treatment, such as the lack of realism of VR scenarios and their suitability for patients, persisted even after the training. For instance, some therapists mentioned that some patients found the environments too artificial, reducing immersion. Others expressed concerns that the available scenarios may not adequately reflect the diverse needs and backgrounds of their patients, limiting their applicability. These concerns are in line with previous research showing that the alignment between technology and patients’ needs is a key factor in the acceptance and sustained use of VR in clinical practice [52]. When technology is tailored to specific patient groups, it is more likely to be perceived as useful, feasible, and engaging, ultimately enhancing treatment experiences and health outcomes [53,54]. Although these concerns persisted, they did not necessarily prevent therapists from using VR. Some therapists noted that as they gained more experience with VR, their initial doubts about realism and suitability became less relevant, particularly when they observed positive patient outcomes. This suggests that while these concerns are valid, their true impact on clinical practice can only be determined through extended use over time. Indicating that implementation and evaluation go hand-in-hand and reinforcing the notion that implementation is an ongoing process, where some barriers can only be addressed once initial adoption has taken place [55,56].

While the training improved therapists’ capability and generally increased motivation to use VR, several opportunity-related challenges hindered the sustained implementation of VR. This aligns with previous studies indicating that organizational factors are underemphasized in implementation efforts, often interrelated with technical and social dimensions, making them complex to address comprehensively [57]. One possible explanation is that many implementation strategies are designed in advance, making it difficult to fully anticipate challenges that only become apparent in later stages of the implementation process. Certain organizational barriers may not initially seem critical but can emerge as important obstacles once therapists attempt to integrate VR into routine practice. This highlights the dynamic nature of implementation and the need for adaptive strategies that evolve based on real-world experiences [56].

In addition, existing organizational theories often fall short of explaining the complexities of health care implementation [58]. As seen in this study, the combined TDF and COM-B model primarily focused on individual factors influencing behavior change, as reflected in the extensive list of determinants within the capability and motivation categories. The COM-B/TDF provided a detailed lens on individual behavior change, which aligned with our interview-based focus on therapists. However, this lens is less focused on organizational dynamics. In CFIR, many COM-B determinants correspond to the *individual characteristics* domain, while CFIR additionally elaborates on the *inner- and outer setting and process*. In our data, several organizational themes emerged that extend beyond COM-B and TDF’s two *opportunity* domains (eg, leadership support, organizational vision, implementation climate, availability of time/rooms/devices, workflow fit). To interpret these layers more fully, future work could combine COM-B and TDF for the behavioral mechanisms with CFIR or NASSS (Nonadoption, Abandonment, Scale-Up, Spread, and Sustainability) to capture contextual determinants related to the organization more comprehensively [40,59]. This also implies broadening data sources beyond therapists to represent organization-level decision processes. Such integration retains TDF’s behavioral focus while clarifying what organizations must put in place for VR to move from training to routine care.

From this longitudinal evaluation, implications for practice emerged. First, findings showed that enthusiasm for VR declined over time due to technical difficulties, limited availability of VR sets, high workload, and time constraints. These findings underscore the importance of ensuring that new technologies are not only functional but also seamlessly integrated into treatment without disturbing established workflows [60]. Despite the extensive training, these issues persisted, indicating that additional layers of support are essential for successful implementation. While training is important for building competence, it is not sufficient on its own to ensure sustained use of innovations. Ongoing technical support, ideally integrated into the workflow, is needed to reduce barriers and facilitate continuous use.

Second, social and hierarchical dynamics within teams influenced implementation. Some therapists felt hesitant to introduce VR as a treatment option, fearing they lacked the authority to promote its use within their team. This suggests that, beyond technical and organizational barriers, workplace culture and professional autonomy play a crucial role in determining whether therapists feel empowered to implement new technologies. Studies have shown that the culture within a health care organization can either promote or hinder innovation. For instance, a study highlighted that cultural values support innovation, but conflicts between existing culture and new ideas can restrain it. The manager’s role is crucial in nurturing this culture, indicating that leadership can significantly influence implementation outcomes [61].

Finally, a lack of organizational vision and sustained support following the training meant that VR was not naturally incorporated into routine treatment practice. Without clear structural reinforcement, such as time allocation, access to equipment, and leadership-driven implementation strategies, therapists were unlikely to prioritize VR over their usual treatment methods. These findings highlight the need for ongoing organizational commitment and support, addressing both technical and cultural barriers, to ensure the successful integration of VR into mental health care [62,63]. It is important to note that the influence of leadership and organizational culture cannot be confined to one-off statements or actions. In this study, it became clear that support from management must be consistently demonstrated and integrated into the ongoing implementation process. It is not enough for leadership to endorse the technology at the outset; they must continue to champion its use, reinforcing that innovation is a priority. This ongoing support is essential to overcome resistance and to ensure that the implementation process is successful in the long term.

These findings indicate that training led to many positive changes in key implementation determinants. However, once the training was completed, new challenges emerged, highlighting the need for additional implementation strategies—particularly those focused on organizational support. A broader and more sustainable implementation process is required for successful VR implementation. Initially, the focus should be on training, but over time, other factors such as organizational support and leadership involvement become more critical. This shift emphasizes the importance of longitudinal evaluation and process monitoring, as challenges evolve. Furthermore, sustaining management’s engagement throughout the process is essential to maintain momentum and address emerging barriers, ensuring long-term success. This aligns with previous research demonstrating that behavior change in clinical practice requires more than training and must include elements of organizational vision, leadership support, and structural facilitation [62,63].

### Strengths and Limitations

This study highlights the value of a longitudinal approach to implementation research, as it captures how key determinants and contextual factors evolve over time. By tracking these changes, it offers practical insights for adapting implementation strategies to real-world dynamics, reinforcing the importance of ongoing evaluation throughout the process [37,64]. Beyond listing determinants, the longitudinal design allowed for interpreting how change unfolded in practice, showing the dynamic interaction between COM-B categories. VR training established capability, capability plus intention to use VR made contextual needs more visible, and opportunity factors ultimately hindered routine use.

The iterative nature of the study also highlights the importance of formative evaluation—not only assessing implementation efforts at the end but also continuously evaluating throughout the implementation process. This enables the identification of changing needs, ensuring that the implementation strategy can be adapted accordingly. Given the scope and the iterative nature of the interviews conducted across 3 timepoints, the sample size was sufficient to capture a comprehensive range of perspectives and experiences, thereby ensuring meaningful insights into the implementation process [65].

In line with recent guidance on reporting qualitative longitudinal research, we make explicit how we handle time and change. Following Audulv et al [66] typology, our results combine a recurrent cross-sectional view per interview round with an integrated, change-focused synthesis to show how patterns shift over time. To support pattern recognition, we used time-ordered bar charts with descriptive counts of coded fragments. These numbers are reported as absolute counts (not percentages) and are meant to complement the thematic interpretation, not to indicate prevalence or allow inferential statistics [67]. They document emerging patterns and should always be read alongside the thematic narrative.

The combined use of TDF and COM-B enabled a systematic analysis of implementation determinants, particularly in distinguishing individual and contextual factors. This supported a deeper understanding of the complexity of VR implementation [34]. However, the use of these models also comes with some limitations. The broad categorization within the capability, motivation, and opportunity categories could oversimplify the complex interactions between these determinants. Specifically, the opportunity category could oversimplify the complex interactions within an organization. Our primary use of individual-level frameworks (COM-B/TDF) may underspecify organizational dynamics. We address this by mapping actionable enablers to CFIR or NASSS in the implications section. Additionally, it can be difficult to clearly distinguish between some determinants in the data, such as between beliefs about capabilities and actual skills. These limitations suggest the need to further refine these models to better capture the dynamic and nuanced nature of implementation processes.

Beyond model-related limitations, this study focused solely on therapists, excluding perspectives from managers, policymakers, trainers, and patients. A broader view could have offered a more comprehensive understanding of implementation determinants [68,69]. Still, focusing on therapists enabled an in-depth exploration of the practical challenges faced by key end users.

Another limitation is limited generalizability, as the study was conducted within a single mental health organization. While this may restrict direct applicability, the insights into how implementation challenges evolve over time are likely relevant across settings. The longitudinal design offers valuable lessons for adapting strategies as implementation progresses. Future research should adopt a multistakeholder, cross-organizational approach to further explore the nuances of VR implementation in diverse contexts [70,71].

This study is a first step in evaluating VR implementation, using an exploratory qualitative longitudinal design to map how therapists’ implementation needs shift over time. Beyond the specific case of VR, it speaks to how time and change can be handled in qualitative longitudinal implementation research. Recent methodological work has noted that temporal aspects in such studies, for example, when changes occur, how stable they are, and what seems to trigger them, are often reported inconsistently, partly because existing guidance on presenting longitudinal findings is limited [66]. Our study offers one pragmatic approach: we made temporal patterns explicit by structuring themes across predefined timepoints and linking them to theory-based constructs (COM-B/TDF), and by using simple counts of coded fragments as descriptive anchors rather than as effect estimates. Future mixed methods implementation studies could build on this approach by pairing qualitative trajectories with repeated quantitative COM-B measures, usage indicators, and selected implementation outcomes as described by Proctor et al [72]. The patterns identified in this study can help inform which determinants to track and how they might be expected to evolve as implementation progresses.

### Conclusions

The implementation strategy of VR training improves the capability of therapists but does not, on its own, lead to sustained use in routine care. Our study showed that organizational and technical challenges posed important barriers to implementation, with the lack of organizational vision and continuous practical support limiting progress. Sustainable implementation, therefore, requires embedding strategies in an iterative, organization-wide implementation approach that targets all layers of implementation: structured follow-up supervision, local champions, protected time and access to rooms and devices, visible leadership engagement, and reliable technical support, aligned with supportive policy and ongoing formative evaluation. The longitudinal design of this study was of great value: it revealed how determinants shifted over time, insights a cross-sectional study would miss, and allowed strategies to be adapted to emerging, dynamic real-world needs.

## Supplementary material

10.2196/83453 Multimedia Appendix 1 Interview Protocols

10.2196/83453 Checklist 1 StaRI (Standards for Reporting Implementation Studies) checklist.

## Abbreviations

CFIR: Consolidated Framework for Implementation Research
COM-B: capability, opportunity, motivation – behavior model
NASSS: Nonadoption, Abandonment, Scale-Up, Spread, and Sustainability Framework
StaRI: Standards for Reporting Implementation Studies
TDF: Theoretical Domains Framework
VR: virtual reality
