# Supplementary material for: Virtual Reality Implementation in Mental Health Care Is a Marathon, Not a Sprint: Qualitative Longitudinal Study of a Virtual Reality Training Program
Source: JMIR Ment Health. 2026 Mar 10;13:e83453. doi: 10.2196/83453 (PMC12974359; doi:10.2196/83453)
Supplement: Multimedia Appendix 1 [file mental-v13-e83453-s001.pdf]

# Appendix A.

## Interview protocol - Round 1

1. Fijn dat je tijd kon vrijmaken en wilt deelnemen aan deze interviews gerelateerd aan VR-scholing.
2. Het doel van deze reeks interviews is om inzicht te krijgen in je verwachtingen en ervaringen met betrekking tot de VR scholing en de inzet van VR in de praktijk. We willen met deze informatie de implementatie van VR verbeteren en alle aandachtspunten uit deze interviews nemen we mee ter verbetering van dit proces.

3. Ik ben als onderzoeker niet persoonlijk betrokken bij de opzet van deze scholing of de uitvoering daarvan. Mijn rol is in die zin heel neutraal en objectief en ik ben alleen geïnteresseerd in eerlijke en open feedback van behandelaren. Voel je dan ook vrij om een kritische blik te geven over de scholing en weet dat ik me niet persoonlijk aangevallen zal voelen.

4. We willen de audio van deze interviews graag opnemen, zodat we je antwoorden kunnen uitschrijven. Alle resultaten worden anoniem verwerkt en zijn niet meer terug te herleiden naar jou als behandelaar Vind je het goed dat we dit gesprek en de komende gesprekken opnemen?

5. Dan vraag ik het je nog een keer, nu de opname aan staat:
    - Ben je akkoord dat we dit gesprek opnemen?
    - Heb je nog vragen of zijn er dingen onduidelijk voordat we het interview starten?

| **Interview 1 – Pre-VR scholing** | |
|---|---|
| **Thema** | **Vragen** |
| Demografische gegevens behandelaar | • Kun je vertellen wat je huidige **functie** is?<br>• Hoeveel jaar **werkervaring** heb je binnen dit vakgebied?<br>• Met welke **doelgroep** werk je en wat voor soort **behandelingen** bied je aan? |
| Ervaring & Mening VR | • Heb je **eerdere ervaringen** met VR? Heb je ooit met deze technologie gewerkt?<br>• Hoe denk je dat VR zou kunnen **bijdragen** aan je werk als behandelaar?<br>• Welke **drempels of barrières** verwacht je die het gebruik van VR in de weg zouden kunnen staan?<br>• In hoeverre heb je het gevoel dat het gebruik van VR **gestimuleerd** wordt vanuit management/organisatie? |
| Verwachting scholing | • **Wat vind je** ervan dat er een scholing wordt georganiseerd voor het gebruik van VR in de behandelpraktijk?<br>• Wat zijn je **verwachtingen** van de VR scholing? |

- Welke specifieke **activiteiten verwacht** je of waar hoop je op?

→ **Globale uitleg opzet scholing**

*VR scholing bestaat uit **6 bijeenkomsten** waarin je met een **groep** leert over de **inzet** van VR, de **toegevoegde waarde**, de verschillende **mogelijkheden** en hoe je dit in een behandeling met cliënten kunt **inzetten**.*
*Je voert elke bijeenkomst **opdrachten** uit en tenslotte zet je een VR behandeling op samen met een **cliënt**. Dit neem je op op **video** en hier schrijf je een **verslag** over.*
*Je werkt samen in een groep, sluit aan bij **intervisiegroepen**, en werkt voor een deel **zelfstandig**.*

- Wat zijn de verwachte **voordelen** van de scholing?

- Wat zijn mogelijke **barrières** of nadelen van de scholing?

- Kan je proberen een aantal **doelen** voor jezelf op te stellen die je wilt bereiken met behulp van deze scholing? (*Deze doelen kunnen we dan in ons laatste interview samen gaan evalueren*)

- Ik wil graag **kennis opdoen of vaardigheden** leren over deze 3 onderwerpen:

- Ik wil X **aantal uur** per week/maand investeren in het leren gebruiken van VR:

- Ik wil graag X **aantal VR sessies** hebben gedaan na 6 maanden in een behandeling met een cliënt:

- Ik wil graag X aantal keer per week/maand **oefenen met een collega:**

- Ik wil graag X aantal keer per maand/jaar deelnemen aan **intervisiegroepen:**

- In hoeverre zijn er punten die voor jou op dit moment **niet duidelijk** zijn rondom de VR scholing of rondom dit onderzoek?

- Heb je verder nog **aanvullingen** die we nog niet besproken hebben tijdens dit interview?

- Dank voor je deelname en succes met de VR scholing! → Over een tijdje zal ik je mailen om het 2e en 3e interview alvast in te plannen, vlak na de scholing en de 3 maanden follow-up.

## Interview protocol - Round 2

1. Dank tijd vrijmaken voor 2e interview.
2. Heel benieuwd naar je ervaringen met de VR scholing en het gebruik van VR in de praktijk.
3. Nogmaals: Voel je vrij om waar nodig ook een kritische blik te werpen en naast positieve ervaringen ook verbeterpunten te noemen.

- In dit interview vraag ik naar je eerste indruk van de VR scholing,
- Ik ga een lijstje met punten af waarover ik graag je ervaringen hoor: zowel positief als negatief
- Vervolgens kijken we of de scholing voldeed aan je verwachting of niet
- Ten slotte kijken we naar de doelen die je hebt opgesteld vorig interview: en of we dit moeten bijstellen of niet.

- Dan vraag ik het je nog een keer, nu de opname aan staat:
  a. Ben je akkoord dat we dit gesprek opnemen?
  b. Heb je nog vragen of zijn er dingen onduidelijk voordat we het interview starten?

| **Interview 2 – post-VR scholing** | **Mid implementation** |
|---|---|
| **Thema** | **Vragen** |
| Eerste indruk scholing | • Kun je je **eerste indruk** van de VR-scholing met me delen?<br>• Wat vond je het meest **opvallend of verrassend**? |
| Positieve punten en verbeterpunten | • Welke aspecten van de scholing vond je **positief** of nuttig?<br>• Zijn er aspecten van de scholing die volgens jou **verbeterd** kunnen worden?<br>1. Inhoud/theorie?<br>2. Werkvormen/oefeningen?<br>3. Inhoudelijke ondersteuning/feedback?<br>4. Technische ondersteuning?<br>5. Leermaterialen? (map, presentatie)<br>6. Inzet praktijk?<br>7. Tijdsplanning?<br>8. Motivatie cliënten?<br>9. Motivatie collega's?<br>10. Intervisiegroep?<br>11. Overig? |
| Klopt de verwachting? | ▪ Hoe verhouden je initiële verwachtingen van de scholing zich tot je **werkelijke ervaring**?<br>• Zijn er aspecten in de scholing aan bod gekomen die je van tevoren **niet had verwacht**?<br>• Heb je **aspecten gemist** in de scholing, die je graag had willen bespreken of willen leren? |
| Verwachting inzet VR | *Je hebt nu kans gehad om VR te ervaren en kort in te zetten in een behandeling. We bespraken vorige keer je verwachtingen van de scholing en we hebben een aantal **persoonlijke doelen** opgesteld.* |

- Ik wil graag **kennis opdoen of vaardigheden** leren over deze 3 onderwerpen:

- Ik wil X **tijd** per week/maand investeren in het leren gebruiken van VR:

- Ik wil graag X **aantal VR sessies** hebben gedaan na 6 maanden in een behandeling met een cliënt:

- Ik wil graag X aantal keer per week/maand **oefenen met een collega:**

- Ik wil graag X aantal keer per maand/jaar deelnemen aan **intervisiegroepen:**

Laten we samen kijken in hoeverre je je **doelen bereikt** hebt.

- In hoeverre heb je je **kennis en vaardigheden** ontwikkeld? Hoe heb je dat gedaan? Heb je daarvoor nog meer nodig ter ondersteuning?

- Heb je het gevoel dat je voldoende **tijd** krijgt/neemt om te investeren in het leren omgaan en inzetten van VR? Waarom wel/niet? Wat heb je daarin nodig?

- **Hoe vaak** heb je VR ingezet in de afgelopen periode? Bij hoeveel (en wat voor soort) **cliënten** heb je VR ingezet?

- Heb je geoefend met **collega's**? Waarom wel/niet? Wat haalde je daaruit?

- Heb je deelgenomen aan **intervisie** bijeenkomsten? Wat haalde je daaruit? En zo nee, waarom niet?

- In hoeverre denk je dat de gestelde doelen **realistisch** zijn? Is het nodig om deze doelen aan te scherpen voor de komende maanden?
  (E.g. aantal cliënten, aantal sessies, wanneer inzetten; specifiekere schatting dan vorige keer)

- Heb je verder nog **aanvullingen**?
  Dank voor je deelname en succes met de inzet van VR! 3e interview in april (dinsdag 9 april 13.30u).

## Interview protocol - Round 3

Fijn dat je tijd kon vrijmaken en wilt deelnemen aan deze interviews gerelateerd aan VR-scholing. Het doel van dit interview is om te kijken hoe je de impact van de VR training ervaart op het behandelen met VR op de langere termijn. Daarnaast kijken we wat voor ondersteuning je nodig zou hebben gehad (of nodig hebt) op het gebied van VR.

Ik ben niet persoonlijk betrokken/ neutraal en objectief /voel je vrij om een kritische blik te geven.

We willen de audio van deze interviews graag opnemen, zodat we je antwoorden kunnen uitschrijven. Alle resultaten worden anoniem verwerkt en zijn niet meer terug te herleiden naar jou als behandelaar Vind je het goed dat we dit gesprek en de komende gesprekken opnemen?

Heb je nog vragen of zijn er dingen onduidelijk voordat we het interview starten?

Dan vraag ik het je nog een keer, nu de opname aan staat:

- Ben je akkoord dat we dit gesprek opnemen?

| **Interview 3 – Interviewschema follow-up** | **Late implementation** |
|---|---|
| **Thema** | **Vragen** |
| **Algemene ervaring & Behalen doelen** | *De vragen in dit interview gaan over de **periode** die begint direct na de VR training tot nu. We beginnen met een algemene vraag en daarna gaan we in wat meer detail kijken.*<br>• In hoeverre **gebruik je VR** op dit moment in je huidige behandelingen?<br>• Heb je een idee **hoe het komt** dat je VR wel/weinig/niet gebruikt? |
| **Behalen doelen** | *Laten we kijken naar de aangescherpte **doelen** die we vorige keer samen gesteld hebben. Vandaag kijken we of deze wel/niet behaald zijn.*<br>• **Kennis/vaardigheden**: wegzakken/afgezwakt?<br>• Ik wil X **tijd** per week/maand investeren in het leren gebruiken van VR<br>• Ik wil graag X **aantal VR sessies** hebben gedaan na 6 maanden in een behandeling met een cliënt<br>• Ik wil graag X aantal keer per week/maand **oefenen met een collega**<br>• Ik wil graag X aantal keer per maand/jaar deelnemen aan **intervisiegroepen** |
| **Motivation** | *De volgende vragen gaan allemaal over je **motivatie** om VR in te zetten in een behandeling en te integreren in je dagelijkse praktijk. We bespreken factoren die van invloed kunnen zijn op die motivatie.* |

- In hoeverre heb je het **gevoel dat je VR kan inzetten**? Voel je je **capabel en comfortabel** genoeg? (M - beliefs about capabilities)
- In hoeverre heb je het idee dat VR van **toegevoegde waarde** kan zijn voor de behandeling met cliënten? (M- beliefs about consequences)
- Vind je dat je VR **zou moeten inzetten** in de behandeling van cliënten? Waarom wel/niet? (M - goals)
- Als je eraan denkt dat je VR moet gaan inzetten in je behandelingen: welk **gevoel** roept dat dan op? En waarom? (M - emotions)
- In hoeverre heb je echt de **intentie** om VR in te gaan zetten in behandelingen met cliënten? Waarom wel/niet? (M - intentions)
- In hoeverre heb jij het idee dat de inzet van VR echt hoort bij jouw **rol/functie** als behandelaar? Is dit een vast onderdeel van je takenpakket? (M- social/professional role/identity)
- In hoeverre **geloof je** dat VR een **standaard onderdeel** gaat worden van de ggz? (M - optimism)
- Jij hebt nu tijd en energie geïnvesteerd in de VR training:<br>In hoeverre **verwacht jij iets terug** te krijgen vanuit de organisatie voor je inzet om je te scholen tot VR behandelaar? Denk bijvoorbeeld aan een certificaat, of extra uren. Denk je dat dit zou helpen bij de inzet van VR?<br>(M - reinforcing behavior)

**Capability**

*De volgende vragen gaan allemaal over of jij het gevoel hebt dat je VR **zou kunnen inzetten** in een behandeling en factoren die daarop invloed kunnen hebben.*

- In hoeverre heb je het gevoel dat je voldoende **kennis en vaardigheden** hebt om VR in te zetten in de behandeling? (C - knowledge/skills)
- In hoeverre denk je aan **VR als behandeloptie** wanneer je een behandeling met een cliënt start? (C - Memory, attention and decision process)
- In hoeverre **plan** je van te voren dat VR een goede optie zou zijn bij een cliënt en **voer je deze plannen dan ook uit**? (C - Behavior regulation)
- Kun je **een specifiek voorbeeld** delen van hoe je VR hebt geïntegreerd in je behandelingen sinds de scholing?

**Opportunity**

*De volgende vragen gaan over **externe factoren** die invloed hebben op jouw VR inzet. Denk aan de invloed van middelen die je tot je beschikking hebt, of de mening van collega's en cliënten.*

- In hoeverre heb je het gevoel dat VR echt **speelt binnen de organisatie** waar je werkt? Is er aandacht voor? Staan mensen ervoor open?

| | |
|---|---|
| | <ul><li>Heb je het gevoel dat je inzet van VR beïnvloed wordt door de **mening of het gedrag** van je collega's, cliënten of management? (O - social influences)</li><li>In hoeverre heb je het **gevoel ondersteund** te worden bij de inzet van VR in je behandelpraktijk? (Vanuit teamleiders, management, collega's)</li><li>In hoeverre heb je het gevoel dat je inzet van VR ligt aan de **middelen** die je tot je beschikking hebt binnen je organisatie? Denk aan voldoende tijd, ruimte, apparatuur, tools/handvaten etc. (O - environmental context/resources)</li></ul> |
| **Verbeterpunten implementatie** | *We hebben nu allemaal factoren besproken die invloed hebben of hebben gehad op het gebruik van VR. Nu wil ik kijken of we nog wat concrete verbeterpunten kunnen bespreken die je nodig hebt, of zou hebben gehad om de inzet van VR te verbeteren.*<ul><li>In hoeverre denk je dat de VR **scholing** heeft bijgedragen aan je **inzet van VR** in de afgelopen periode?</li><li>Wat is er nog **meer nodig** om je ondersteuning te bieden om VR in de praktijk te gebruiken? Welke **randvoorwaarden** zijn er?</li><li>**Door wie** moet dat geregeld worden/Wie zou daar **verantwoordelijk** voor moeten zijn?</li></ul> |
| **Verwachting toekomst** | *Als we kijken naar de toekomst…*<ul><li>Verwacht je VR zelf te **blijven inzetten** in behandelingen met cliënten? Waarom wel/niet?</li><li>Heb je **overige punten** die je graag wilt bespreken? Heb je het gevoel dat we nog iets niet besproken hebben wat van belang is bij de implementatie van VR in de praktijk?</li></ul> |
